# Application of Residual Structure Time Convolutional Network Based on Attention Mechanism in Remaining Useful Life Interval Prediction of Bearings

**DOI:** 10.3390/s24134132

**Published:** 2024-06-26

**Authors:** Chunsheng Zhang, Mengxin Zeng, Jingjin Fan, Xiaoyong Li

**Affiliations:** 1 SCNU Environmental Research Institute, Guangdong Provincial Key Laboratory of Chemical Pollution and Environmental Safety & MOE Key Laboratory of Theoretical Chemistry of Environment, School of Environment, South China Normal University, Guangzhou 510006, China; 2 Shantou Yerei Technology Co., Ltd., Shantou 515000, China; 3 Shanwei Institute of Technology, Shanwei 516600, China; 4 Research Institute of History for Science and Technology, Nanjing University of Information Science & Technology, Nanjing 210044, China

**Keywords:** rolling bearings, remaining useful life prediction, attention mechanism, residual structure, temporal convolution, interval prediction

## Abstract

In the context of Industry 4.0, bearings, as critical components of machinery, play a vital role in ensuring operational reliability. The detection of their health status is thus of paramount importance. Existing predictive models often focus on point predictions of bearing lifespan, lacking the ability to quantify uncertainty and having room for improvement in accuracy. To accurately predict the long-term remaining useful life (RUL) of bearings, a novel time convolutional network model with an attention mechanism-based soft thresholding decision residual structure for quantifying the lifespan interval of bearings, namely TCN-AM-GPR, is proposed. Firstly, a spatio-temporal graph is constructed from the bearing sensor signals as the input to the prediction model. Secondly, a residual structure based on a soft threshold decision with a self-attention mechanism is established to further suppress noise in the collected bearing lifespan signals. Thirdly, the extracted features pass through an interval quantization layer to obtain the RUL and its confidence interval of the bearings. The proposed methodology has been verified using the PHM2012 bearing dataset, and the comparison of simulation experiment results shows that TCN-AM-GPR achieved the best point prediction evaluation index, with a 2.17% improvement in R^2^ compared to the second-best performance from TCN-GPR. At the same time, it also has the best interval prediction comprehensive evaluation index, with a relative decrease of 16.73% in MWP compared to the second-best performance from TCN-GPR. The research results indicate that TCN-AM-GPR can ensure the accuracy of point estimates, while having superior advantages and practical significance in describing prediction uncertainty.

## 1. Introduction

Bearings are crucial mechanical elements used to support and reduce friction between rotating parts in mechanical devices. They play an essential role in various industrial applications and mechanical systems such as the automotive industry, medical equipment, and aerospace, ensuring the reliability, efficiency, and durability of equipment. Damage to bearings can affect the performance and lifespan of equipment and may lead to severe safety risks. Predicting the remaining useful life (RUL) of bearings aims to estimate how much time or how many working cycles remain in a bearing’s future service life, to determine equipment maintenance schedules and avoid unexpected machine failures. Generally, the existing prediction methods can be categorized into the following three types: statistical learning, machine learning, and deep learning.

Predictions of bearing RUL based on statistical methods typically rely on the statistical analysis of bearing monitoring data to identify features and patterns associated with bearing failure. Common approaches include time-series analysis, box plots and scatter plots, Weibull distribution, Log-Normal distribution, Kaplan–Meier survival curves, Cox proportional hazards model, Bayesian statistics, etc. However, these techniques require extensive monitoring data and statistical analysis. Specifically, they demand high data quality and are unable to handle complex working conditions such as sudden failures, significantly limiting the model’s applicability. In contrast, bearing RUL prediction algorithms based on machine learning offer superior nonlinear modeling and feature learning capabilities compared to statistical methods. Examples include Support Vector Machines (SVMs), Random Forests, Decision Trees, Bayesian Networks, AdaBoost, etc.

Deep learning-based technologies have significantly advanced fields such as computer vision and natural language processing due to their capability to process complex, nonlinear data, attracting increasing attention from scholars. The use of convolutional neural networks (CNNs) for predicting bearing life has made notable progress in many studies. For instance, Chen et al. [1] achieved a good predictive performance by automatically extracting features from vibration signals using CNNs for mechanical fault diagnosis, including bearing life prediction. Li et al. [2] focused on bearing fault diagnosis in noisy environments and utilized CNNs to process vibration signal data, demonstrating the CNN’s robustness in handling noisy data. Che et al. [3] employed a pretrained CNN model based on transfer learning to adapt to bearing fault detection under different operating conditions, showcasing the versatility of CNNs across various operational scenarios. However, the reliance on large amounts of labeled data, which might be difficult to obtain, and overlooking the temporal dependencies in time-series data limit the applicability of CNNs.

Although Spatio-Temporal Networks (STNs) are relatively new, they have the ability to handle the temporal dependencies of time-series data, introducing attention mechanisms to prediction models and thereby enhancing predictive performance. Huang et al. [4] proposed a two-stage bearing life prediction model, where the first stage utilizes a Deep Spatio-Temporal Attention Network (STAN) to extract features that capture temporal relationships in time-series data. The second stage employs Random Forest to integrate features for life prediction. Zhu et al. [5] introduced a method based on a feature fusion network that includes spatio-temporal features extracted using CNNs and raw mapping features obtained using a fully connected layer. Zhang [6] developed a dual-channel feature fusion network that utilizes STNs’ temporal and spatial attention to capture key information in the data for bearing life prediction. STNs, by integrating spatio-temporal attention mechanisms, can better capture the temporal and spatial relationships in data. However, their complexity is higher, potentially requiring more computational resources and training time.

The CNN-LSTM architecture leverages the strengths of CNNs in extracting spatial features from time-series data while utilizing LSTMs to model the temporal dependencies within sequential data, resulting in an outstanding performance in predicting bearing life. This approach similarly requires a substantial amount of labeled data for training, and the complexity of the model can be relatively high. Gao et al. [7] proposed using CNNs to extract features from vibration signals, which are then fed into an LSTM to model the temporal dependencies of the time-series data for predicting the RUL of bearings. Similar efforts by Li et al. [8], Zheng et al. [9], Zhang et al. [8], and others have showcased the advantages of combining CNNs and LSTMs to enhance bearing life prediction performance. However, these methods face potential challenges such as computational complexity and the need for hyperparameter tuning.

Therefore, this study proposes a bearing life prediction model based on Temporal Convolutional Networks (TCNs) and attention mechanisms (AMs). TCNs are an emerging deep learning architecture combining convolutional operations with temporal dependency modeling. Its parallelism and scalability characterize it, making it suitable for processing time-series data, such as bearing vibration signals. TCNs are capable of capturing both long-term and short-term temporal dependencies without the need for the long-term memory associated with Recurrent Neural Networks (RNNs). The attention mechanism allows the model to dynamically focus on important information when processing input data, thereby improving the model’s accuracy. In the context of bearing life prediction, it can assist the model in automatically determining which signals or features are more important.

RUL prediction also faces another challenge, as current major works are based on point estimate algorithm models. Under complex operating conditions, RUL prediction can be influenced by various factors, including sensor noise, modeling uncertainty, and the random changes of future environments and conditions. These factors reduce the credibility of point estimate methods. Therefore, it is necessary to delve into how to quantify the uncertainty in RUL prediction. Lee et al. [10] use Gaussian Process Regression (GPR) for probabilistic prediction of bearing life. GPR can capture the uncertainty of bearing life, providing a probability distribution rather than just a point estimate. Yuan et al. [11] employ Quantile Regression (QR) for bearing life prediction, which allows for the direct estimation of the quantiles of bearing life rather than just the mean. This approach is particularly helpful in capturing the uncertainty of life prediction and can provide more comprehensive information. He et al. [12] use Kernel Density Estimation (KDE) for bearing life prediction. KDE is a non-parametric method that can estimate the probability density function of bearing life. Compared to the above methods, GPR has advantages in uncertainty modeling, wide applicability, flexibility of kernel functions, sample efficiency, and interpretability. Therefore, this paper explores a bearing life interval prediction model based on GPR to further enhance the quantification ability to bear uncertainty.

The main contributions of this paper are as follows: (1) an improved TCN was proposed, which leverages the convolutional operations and temporal dependency features of TCN for bearing life prediction; (2) the TCN network based on the post-attention mechanism enhances the point prediction performance of the model; (3) by integrating the interval prediction functionality of GPR, TCN-AM-GPR is proposed to improve the quantification of uncertainty in bearing life prediction results.

The remainder of this paper is organized as follows: Section 2 covers the preliminary work. The methodology framework proposed in this paper is presented in Section 3, including the construction of the TCN network, the design of the self-attention residual structure mechanism model, and the GPR interval prediction method. In Section 4 and Section 5, several comparative experiments using bearing datasets are conducted to demonstrate the superiority of TCN-AM-GPR. Finally, Section 6 concludes the paper.

## 2. Theoretical Background

### 2.1. Temporal Convolutional Network

TCNs are a novel deep learning network designed specifically for solving time-series problems [13]. They comprise three key components [14], as follows: causal convolution, dilated convolution, and residual connections. Causal convolution ensures that the model can only use information from the past, while dilated convolution effectively captures long-term dependencies [15]. Moreover, TCNs employ a fully convolutional network structure, capable of handling input sequences of varying sizes and ensuring that the output size matches the input sequence size [16]. This makes TCNs exceptionally performant in addressing issues such as time-series prediction.

Causal convolution is a type of convolutional operation that strictly follows the sequence of time [17]. Its distinguishing feature is that it calculates the value of the current moment based solely on the data from the current and previous moments, without being influenced by any future data. Unlike traditional CNNs, causal convolution networks are unable to use future data for predicting current outcomes, thereby adhering strictly to temporal sequence constraints [18].

Dilated convolution is a type of convolutional operation that expands the receptive field by skipping input data [19]. Unlike traditional CNNs, dilated convolution introduces a dilation factor, allowing the network’s scope of receiving historical information to be freely adjusted by changing the size of the dilation factor. For a one-dimensional input sequence, $x\in R^{n}$, and a filter, f:{0,⋯,k−1}→R, the operation of dilated convolution can expand the receptive field through the filter, k, and dilation, d, with the dilated convolution operation defined as follows:

(1) $$F_{s}=\sum \left(k*s_{di}\right)$$

where s represents the current data, and $s_{\mathrm{di}}$ denotes the historical data in the input sequence.

**Figure 1:**
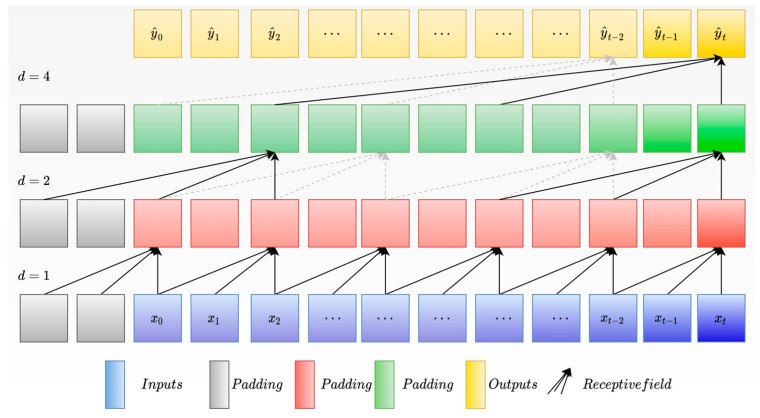
Structure of the dilated causal convolution.

The dilated causal convolution is illustrated in Figure 1, which shows examples of different dilation factors (d=1,2,4) and filter sizes (k=3). It can be observed that the receptive field of the output sequence, $y_{t}$, can be flexibly changed by adjusting k and d, and the output results are only influenced by previous historical data. Through this method, we can flexibly adjust the receptive field to fully consider the temporal characteristics of electric load data. Depending on the input time scale, we can adjust the memory length of the output nodes, effectively solving the problem of forgetting historical data that is present in traditional methods, and making it more suitable for bearing life prediction.

Residual connections enable the network to transmit information in a cross-layer manner, effectively solving problems such as gradient vanishing and gradient explosion [20]. The residual connection block is shown in the figure. The TCN consists of two residual connection blocks, which add the input and the output learned by the network through the residual connections, and then serve as the input for the next residual block. The residual connection can be represented as follows:

(2) O=Activation(x+F(x))

where O is the output, F(⋅) represents a series of branches learned by the network, and x is the input. This mechanism helps to alleviate gradient issues during the training process and aids the network in improving learning and optimization processes.

Therefore, TCNs can achieve large-scale parallel processing when handling data. By adjusting the filter size, k, and dilation factor, d, it can flexibly control the size of the receptive field of the output, thereby regulating the memory length of the model and better considering the temporal characteristics between data. These features enable TCNs to exhibit exceptional performances in processing time-series data.

### 2.2. Attention Mechanism

**Figure 2:**
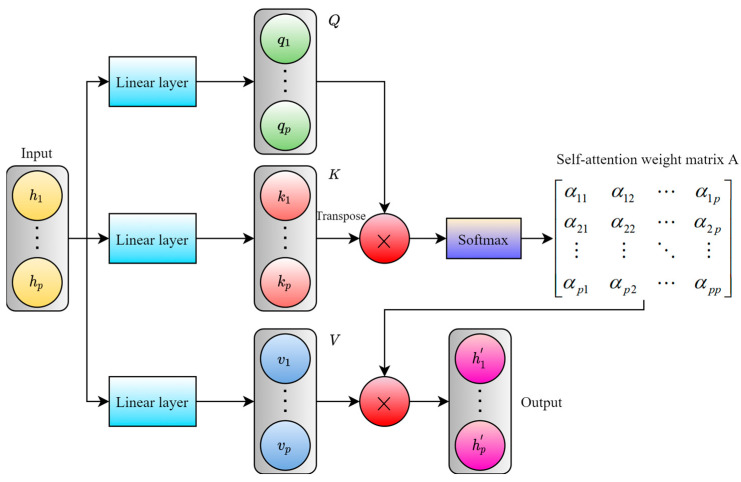
The structure of the self-attention mechanism.

The self-attention mechanism is a crucial technique in deep learning, initially applied to natural language processing tasks such as machine translation [21]. It can capture the dependency relationships between different positions when processing sequence data, allowing the model to dynamically allocate attention weights to different positions [22]. This enables a better understanding of the important information within the sequence [23]. The calculation process structure is shown in Figure 2.

First, the input data are processed through three linear layers to generate the query matrix, Q; key matrix, K; and value matrix, V. Next, by multiplying the query matrix, Q, with the transpose of the key matrix, K, and dividing by a scaling factor, the softmax function is applied to calculate the self-attention weight matrix, A. Finally, the value matrix, V, is multiplied by the self-attention weight matrix, A, to obtain the output result weighted using the self-attention mechanism. Alternatively, two linear layers can be used to generate the query matrix, Q, and key matrix, K, and the input can be directly multiplied by the weight matrix, A, to obtain the output. The calculation process can be represented using the following equation

(3) $$attn(h)=softmax\left(\frac{QK^{T}}{\sqrt{d_{k}}}\right)V$$

where $d_{k}$ is the scaling factor, which is used to prevent the results of the matrix multiplication from becoming too large. attn(h) represents the input h weighted using the self-attention mechanism, i.e., the output result of the self-attention mechanism.

### 2.3. Gaussian Process Regression

**Figure 3:**
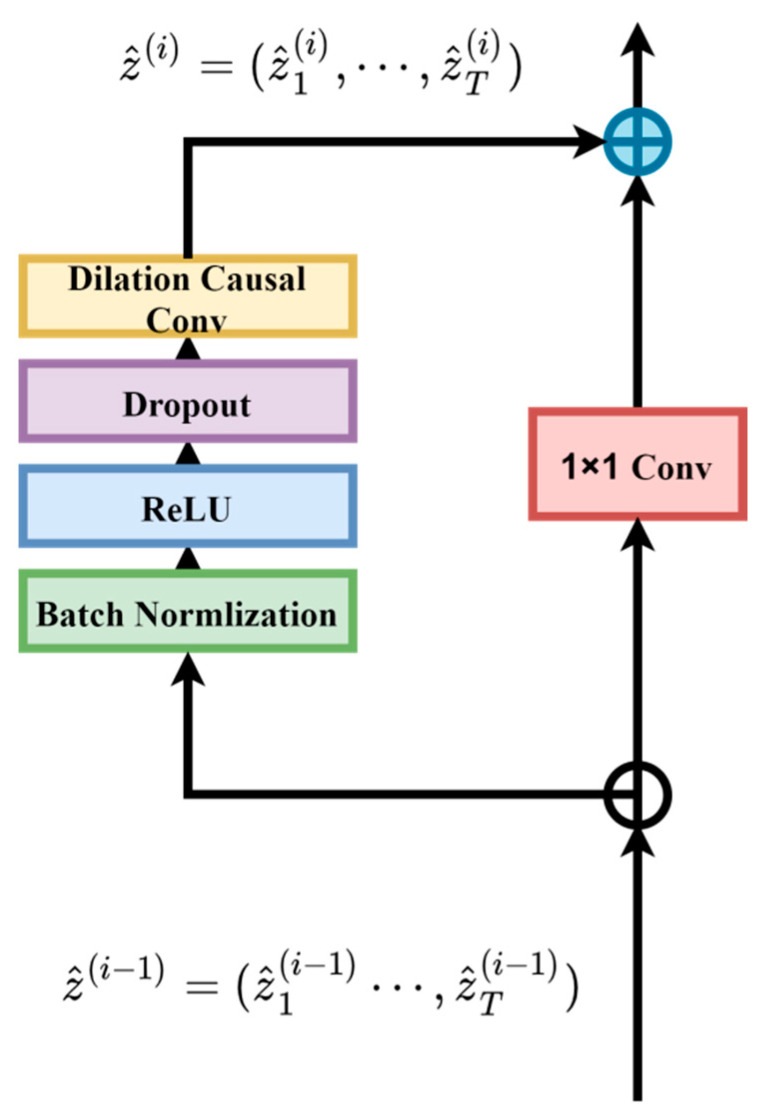
Schematic diagram of traditional residual structure.

**Figure 4:**
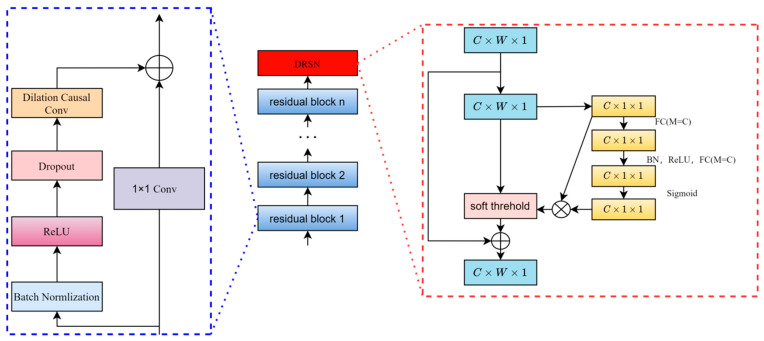
Residual structure with integrated soft thresholding via attention mechanism.

Compared to the traditional residual structure [24] shown in Figure 3, which only involves residuals between consecutive convolutional layers, the improved residual structure proposed in this study is illustrated in Figure 4. The entire structure includes shallow CNN modules, deep CNN modules, residual structures, attention modules, and fully connected and regression modules. The shallow CNN module comprises convolutional layers, batch normalization layers, activation layers, pooling layers, and dropout layers. The residual structure fuses the features output by the shallow CNN module with those from the deep CNN module. The attention module then filters these fused features.

### 2.4. Gaussian Process Regression

GPR is a method suitable for handling nonlinear data, utilizing the properties of joint Gaussian distributions to model data relationships [25]. Consider a sample dataset represented as $D=\left(x_{i},y_{i}\right)$, in which i∈{1,2,⋯,n}, $x_{i}$ is the input vector, and $y_{i}\in R$ is the corresponding output value. Let X denote the input matrix; then, the standard linear regression model with Gaussian white noise is given by [26]:

(4) y=f(X)+ε

where ε is an independent random variable, following a Gaussian distribution with mean, 0, and variance, $\sigma^{2}$, denoted as $\varepsilon ∼N\left(0,\sigma^{2}\right)$.

From Equation (4), the prior distribution of the observed target value, y, is a Gaussian distribution, represented as [26]:

(5) $$y∼N\left(0,C+\sigma_{n}^{2}I\right)$$

For a new test input, $x^{*}$, the joint Gaussian distribution formed between the observed values, y, of the training samples and the input variable, $y^{*}$, of the test data is as follows:

(6) $$\left[\begin{array}{c} y\\ y^{*} \end{array}\right]∼N\left(0,\left[\begin{array}{cc} C(X,X)+\sigma_{n}^{2}I & C\left(X,X^{*}\right)\\ C\left(X^{*},x^{*}\right) & C\left(x^{*},x^{*}\right) \end{array}\right]\right)$$

where $C\left(X,x^{*}\right)=C{\left(x^{*},X\right)}^{T}$ is the covariance matrix between the test data, $x^{*}$, and the inputs, X, of the training set, representing the covariance matrix of $x^{*}$ itself.

Given the test input, $x^{*}$, and the training set, D, the goal of Gaussian Process Regression is to determine the corresponding form through the posterior probability formula [26], namely:

(7) $$\begin{array}{c} y^{*}|x^{*},D∼N\left(\mu_{y^{*}},\sigma_{y^{*}}^{2}\right)\\ \mu_{y^{*}}=C\left(x^{*},X\right){\left(C(X,X)+\sigma_{n}^{2}I\right)}^{-1}\\ y=\sum_{i=1}^{n}\alpha_{i}C\left(x_{i},x^{*}\right) \end{array}$$

where $\mu_{y^{*}}$ and σ represent the expectation and variance of $y^{*}$, respectively, and $\alpha ={\left(C+\sigma_{n}^{2}I\right)}^{-1}y$, where I is the identity matrix of order n.

According to Equation (7), the covariance function of GPR is essentially the kernel function used by traditional machine learning models. By mapping nonlinear data into feature space, the GPR model seeks the linear relationships between data, thereby transforming the originally complex nonlinear problem into a simpler linear problem. In the GPR model, different covariance functions can be chosen to suit different data characteristics. This paper adopts various single covariance functions, including the Squared Exponential covariance function (SE), the Rational Quadratic covariance function (RQ), and the Matérn covariance function, and combines them into a kernel function to better model the relationships between data.

## 3. Methodology

**Figure 5:**
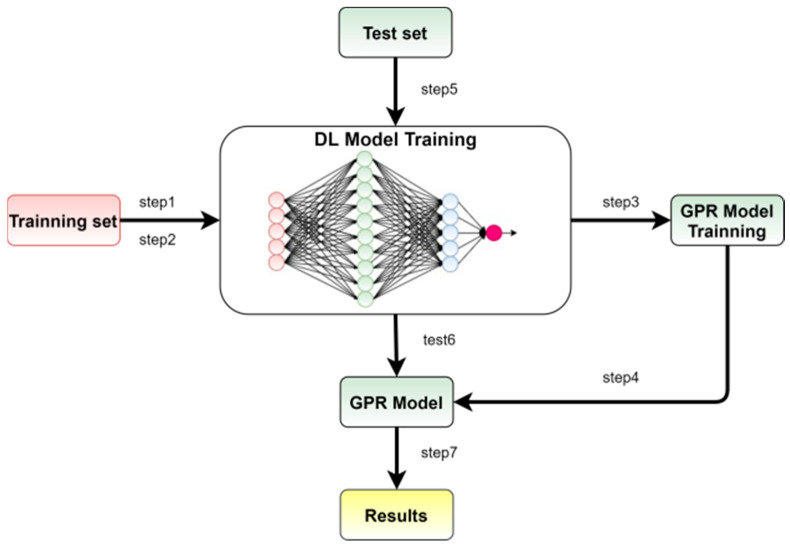
Training and testing process of the TCN-AM-GPR prediction model.

The approach to obtaining high-precision point predictions, reliable interval predictions, and probability predictions involves combining the advantages of point predictions using Temporal Convolutional Networks, with a soft threshold residual structure based on the attention mechanism, and the advantages of GPR interval predictions. This approach leads to the proposal of the TCN-AM-GPR model for RUL interval prediction. The training and testing processes of the TCN-AM-GPR model are illustrated in Figure 5.

The specific process of this method is as follows: Firstly, the TCN-AM-GPR model is trained in the single-point prediction process, corresponding to step 1 in Figure 5. Secondly, the training values are input into the trained CTA-net model to obtain outputs (corresponding to step 2 in Figure 5), and this output is used as the training sample for GPR (corresponding to step 3 in Figure 5). At the same time, the labels corresponding to the original training set are used as the labels for GPR, and the GPR model is trained (corresponding to step 4 in Figure 5). The test set is input into the TCN-AM-GPR model (corresponding to step 5 in Figure 5) to obtain single-point prediction values (corresponding to step 6 in Figure 5), and then this single-point prediction value is input into the GPR model (corresponding to step 7 in Figure 5) to obtain the interval prediction value. Its advantages mainly include two aspects, as follows: on the one hand, it inherits the high accuracy of the CTCN-AM-GPR model in single-point prediction; on the other hand, based on the high accuracy of the TCN-AM-GPR model in point measurement, when constructing GPR between the first point measurement value and the observed value, a more reliable interval prediction range and PDF can be obtained.

## 4. Experiment Data

### 4.1. Databases

**Figure 6:**
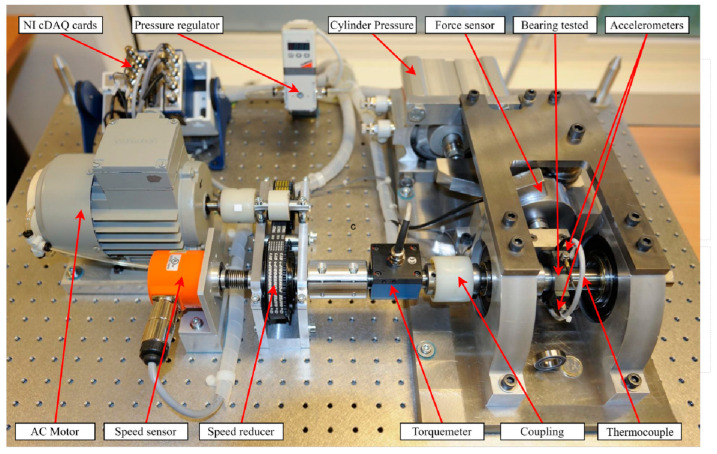
Bearing acceleration degradation PRONOSTIA test bench.

To validate the generality and effectiveness of the interval prediction method based on a CNN, we selected the bearing acceleration life experimental data provided by the IEEE PHM 2012 Challenge for validation analysis [27]. These experimental data originate from the PRONOSTIA test bench, as shown in Figure 6. There are two accelerometers installed on the bearing housing, used to measure vertical and horizontal vibrations, respectively. The sampling interval of these data is 10 s, with a sampling rate of 25.6 kHz, and each sampling lasts for 0.1 s, obtaining 2560 data points per sampling [28].

### 4.2. Evaluation Metric for Experimental Results

#### 4.2.1. Point Prediction Evaluation Metrics

In this paper, the Mean Absolute Error (MAE), Root Mean Square Error (RMSE), and the Coefficient of Determination (denoted as R^2^) are selected as the three evaluation metrics to assess the performance of the point prediction methods [29], as shown in Equations (8), (9) and (10), respectively.

(8) $$MAE=\frac{1}{n}\sum_{i=1}^{n}\left|\left(p_{i}-y_{i}\right)\right|$$

(9) $$RMSE=\sqrt{\frac{1}{n}\sum_{i=1}^{n}(y_{i}-p_{i}{)}^{2}}$$

(10) $$R^{2}=1-\frac{\sum_{i=1}^{n}\left(p_{i}-\bar{y}\right)}{\sum_{i=1}^{n}\left(y_{i}-\bar{y}\right)}$$

wherein *n* represents the number of test samples; $\bar{y}$ is the average of the actual target values, $y_{i}$; and $p_{i}$ is the predicted value. The smaller the values of RMSE and MAE, the better the point prediction performance of the model is considered to be, while a value closer to 1 indicates that the model’s point prediction results have a higher degree of fit with the observed values.

#### 4.2.2. Interval Probability Prediction Evaluation Metrics

In this paper, the interval prediction metrics include Coverage Probability ($CP_{\alpha }$), Mean Width Percentage ($MWP_{\alpha }$), Mean Width Coverage ($MC_{\alpha }$), and Probability Integral Transform (PIT), as shown in Equations (11)–(14). Here, α represents the confidence interval, with the commonly used 95% confidence interval being selected for this study [29].

(11) $$CP_{\alpha }=\frac{c_{\alpha }}{T_{e}}$$

(12) $$MWP_{\alpha }=\frac{1}{T_{e}}\sum_{i=1}^{T_{e}}\frac{up_{i}-down_{i}}{y_{i}}$$

(13) $$MC_{\alpha }=\frac{MWP_{\alpha }}{CP_{e}}$$

(14) $$PIT=\int_{-\infty }^{Y_{i}}p(x)dx$$

### 4.3. Comparison Methods

In this experiment, five deep learning prediction models were established, as follows: CNN, LST, CLSTM, TCN, and TCN-AM. Among these, CLSTM represents a model that directly connects and merges CNNs with LSTMs (all using consistent CNN and LSTM structures, with the output of CNNs directly serving as the input for LSTMs). The TCN is a model that adopts dilated convolution on the basis of CNNs and incorporates a residual structure. TCN-AM is the model constructed in this study, differing from TCN in that it employs a residual structure with a soft threshold attention mechanism for feature selection, and it compares the constructed model from various aspects.

## 5. Experiment and Analysis

### 5.1. Experimental Environment

In this study, all models were trained and performed prediction classifications on a computer with the same configuration, namely a CPU of Core i9-10900 K (Intel, Santa Clara, CA, USA), 32 GB of RAM, and a GeForce RTX 3080Ti graphics card (NVIDIA, Santa Clara, CA, USA). The operating system running on the computer was Windows 10 Professional, and all models were implemented using Matlab (version 2023b). This setup ensures that all models are compared under the most similar conditions possible, facilitating a comprehensive reflection of the true performance of the CNN, LSTM, CLSTM, TCN, and TCN-AM models, as well as the models combined with GPR.

### 5.2. Model Parameter Settings

During the model training process, the parameter settings commonly used in training deep learning models were referenced. The Kaiming method was used to initialize the weights of the CNN layers and fully connected layers [30], and the orthogonal method was used to initialize the weights of the attention layers [31]. The Adam optimizer was used during the network training process to make the model converge faster and be more robust, while also reducing the validation set testing [32]. The experimental data utilized the PHM 2012 bearing degradation dataset, which involves three different operating conditions [33], and the division between the training set and test set has already been completed, as shown in Table 1.

### 5.3. Analysis of Experimental Results

This study conducts simulation experiments on the PHM2012 dataset, testing the interval prediction method based on TCN-GPR. The experiments use the training and test sets for Operating Condition 1 in Table 1, selecting the *x*-axis vibration data for analysis. In Operating Condition 1, the bearing’s speed is kept constant at 1800 rpm, and bearing damage is accelerated by applying a radial load of 4000 N; if the accelerometer’s amplitude exceeds 20 g, the bearing is considered to have failed, and data collection is stopped.

**Figure 7:**
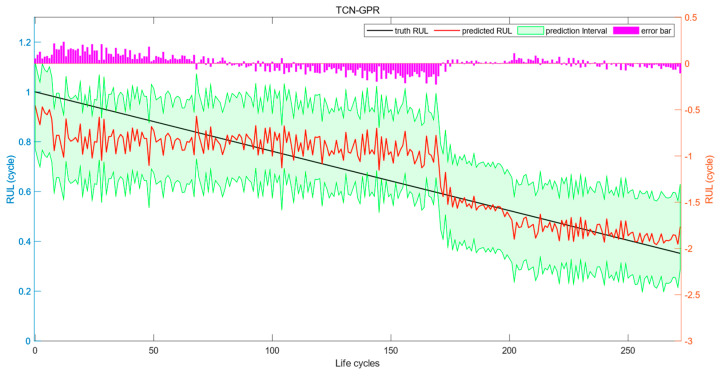
Interval prediction results of TCN-GPR.

For the test set, the interval prediction results of TCN-GPR are shown in Figure 7. As is shown in the figure, the black line represents the actual RUL values, the red line shows the point prediction results for RUL, the green shaded area describes the interval prediction results quantifying data uncertainty, and the purple bar indicates the error between the actual RUL values and the point prediction results. In the experiment shown in Figure 6, the point prediction of RUL has an MAE value of 0.0665, an RMSE value of 0.0853, and an R^2^ value of 0.7937. The interval prediction has a CP value of 0.9487, an MWP value of 0.5507, and an MC value of 0.5805.

**Figure 8:**
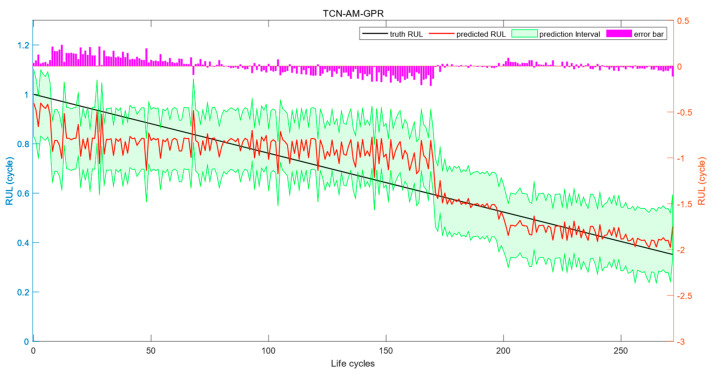
Interval prediction results of TCN-AM-GPR.

For the test set, the interval prediction results of TCN-AM-GPR are shown in Figure 8. As is shown in the figure, the point prediction of RUL has an MAE value of 0.0621, an RMSE value of 0.0817, and an R^2^ value of 0.8109. The interval prediction has a CP value of 0.8571, an MWP value of 0.4144, and an MC value of 0.4834. In the test set, TCN-AM-GPR shows a clear advantage in point prediction performance over TCN-GPR, with reductions in MAE and RMSE values by 6.62% and 4.42%, respectively, and an increase in the R^2^ value by 2.17%; although the CP value decreased in interval prediction performance, the MWP value shows a significant advantage, and the comprehensive evaluation metric CM decreased by 16.73%.

**Figure 9:**
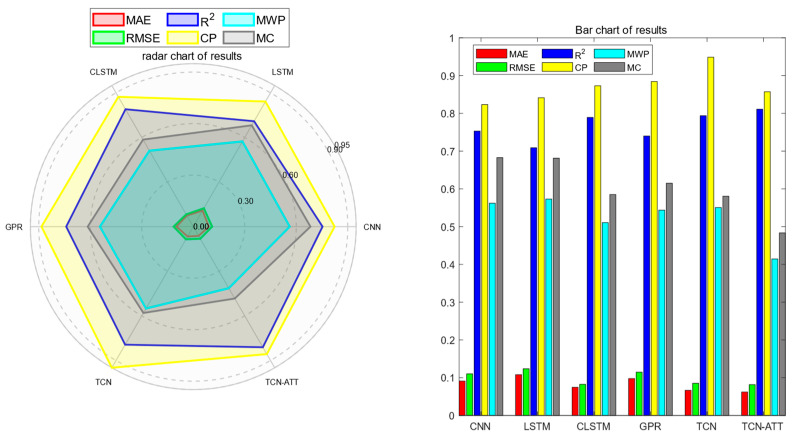
Visualization of evaluation metrics for prediction results.

The evaluation metrics for the simulation results of all comparative models can be summarized in graphs, including radar charts and bar charts (as shown in Figure 9); the results indicate that the TCN-AM-GPR method, an improvement based on TCN-GPR, achieves a relatively high accuracy in point predictions, and its interval prediction results provide a more meaningful range for guiding actual maintenance strategies.

Additionally, a summary of the interval prediction results for deep learning models with similar structures is shown in Table 2. As shown in Table 2, the interval prediction method based on TCN-AM, while ensuring the effectiveness of point predictions, can more accurately describe the uncertainty during the bearing degradation process, exhibiting a superior overall performance compared to classical uncertainty quantification methods.

## 6. Conclusions

This study presents a time convolutional network that integrates an attention mechanism and a soft threshold residual structure, combined with Gaussian Process Regression (GPR) for interval prediction of bearing lifespan, demonstrating a good performance that is validated using the PHM2012 bearing degradation public dataset. The model features are as follows: (1) Better feature extraction capability: the TCN network can capture long-term dependencies in sequence data, effectively extracting important features from the sequence. The integration of the attention mechanism and soft threshold residual structure further enhances this capability; (2) Efficient modeling capacity: the TCN network has fewer parameters and computational requirements, reducing model complexity and training time, while maintaining high modeling capability; (3) Integration of GPR advantages: combining the TCN-AM network with GPR allows for the integration of features learned by the TCN network with the Gaussian process regression model of GPR, thereby providing more accurate mechanical life interval predictions. The current method primarily models and predicts using single-modality data. In the trial set used for actual simulation testing, TCN-AM-GPR showed significant advantages in the performance of point prediction and interval prediction compared to TCN-GPR. The evaluation index R^2^ value of point prediction increased by 2.17%, and the comprehensive evaluation index CM of interval prediction decreased by 16.73%. Future work could consider integrating data from multiple modalities, such as structure, vibration, temperature, etc., to improve prediction accuracy. A more refined classification mechanism can be implemented to achieve RUL prediction of engineering systems under different operating conditions and fault modes.

## Figures and Tables

**Table 1:** PHM 2012 bearing degradation dataset.

Dataset	Working Condition 1	Working Condition 2	Working Condition 3
Training set	Bearing1_1	Bearing2_1	Bearing3_1
	Bearing1_2	Bearing2_2	Bearing3_2
Testing set	Bearing1_3	Bearing2_3	Bearing3_3
	Bearing1_4	Bearing2_4	-
	Bearing1_5	Bearing2_5	-
	Bearing1_6	Bearing2_6	
	Bearing1_7	Bearing2_7	-

**Table 2:** Predictive performance metrics of all comparison models on the test set.

Model	MAE	RMSE	R^2^	CP	MWP	MC
CNN	0.0911	0.1101	0.7532	0.8233	0.5621	0.6827
LSTM	0.1081	0.1237	0.7088	0.8412	0.5731	0.6813
CLSTM	0.0745	0.0824	0.7893	0.8730	0.5108	0.5851
GPR	0.0978	0.1147	0.7401	0.8843	0.5438	0.6149
TCN	0.0665	0.0853	0.7937	0.9487	0.5507	0.5805
TCN-ATT	0.0621	0.0817	0.8109	0.8571	0.4144	0.4834

## Data Availability

The original contributions presented in the study are included in the article, further inquiries can be directed to the corresponding author.
